# Physiotherapy students can be educated to portray realistic patient roles in simulation: a pragmatic observational study

**DOI:** 10.1186/s12909-020-02382-0

**Published:** 2020-11-26

**Authors:** Shane A. Pritchard, Jennifer L. Keating, Debra Nestel, Felicity C. Blackstock

**Affiliations:** 1 Department of Physiotherapy, Monash University, Moorooduc Highway, Frankston, Victoria 3199 Australia; 2 Monash Institute for Health and Clinical Education (MIHCE), Monash University, Clayton, Victoria Australia; 3 Department of Surgery, University of Melbourne, Parkville, Victoria Australia; 4 School of Science and Health, Western Sydney University, Campbelltown, New South Wales Australia

**Keywords:** Simulation, Physiotherapy, Simulation-based education, Peer simulation, Simulated patient, Standardized patient, Physical therapy

## Abstract

**Background:**

Simulation-based education (SBE) has many benefits for learners, but costs can limit embedding SBE in health professional curricula. Peer simulation involves students portraying patient roles, and may reduce costs while still providing the benefits of other SBE experiences. However, the quality of the SBE may be impacted if students cannot portray authentic and realistic patient roles. The aim of this study was to investigate whether targeted education was associated with observable changes to physiotherapy students’ abilities to portray patient roles in SBE.

**Methods:**

Second year pre-registration physiotherapy students (*n* = 40) participated. Students completed online and face-to-face education about SBE, patient portrayal skills, and how to portray a specific patient role. Students were video-recorded portraying patient roles in practical exams before and after the program. Three blinded independent assessors rated the overall quality of portrayals using a purpose-developed assessment instrument.

**Results:**

Twenty-three sets of pre- and post-program videos were analysed. Correlations between assessor scores spanned 0.62 to 0.82 for analyses of interest, which justified using average assessor ratings in analysis. Statistically significant higher scores were seen for post-program assessments for overall portrayal scores (mean difference 6.5, 95%CI [1.51–11.45], *p* = 0.013), accuracy (mean difference 3.4, 95%CI [0.69–6.13], *p* = 0.016) and quality (mean difference 3.1, 95%CI [0.64–5.49], *p* = 0.016).

**Conclusions:**

Physiotherapy students appear capable of playing realistic patient roles. Peer simulation can be embedded into health professional programs, and education in patient role portrayal appears to be associated with improvements in portrayal quality and realism. Given these findings, further investigation, including testing program effects in a randomised study, is warranted.

**Supplementary Information:**

The online version contains supplementary material available at 10.1186/s12909-020-02382-0.

## Background

Simulation-based education (SBE) refers to learning and teaching activities for health professional students that “replace or amplify real experiences with guided experiences, [and] evoke or replicate substantial aspects of the real world in a fully interactive manner” [1]. It typically involves simulators (e.g. task trainer, manikin, virtual patient) or people (simulated/standardized patients (SPs), confederates) situated in simulated clinical environments that enable learners to perform clinical tasks as if they were interacting with a real patient [2, 3].

SBE is highly effective for improving health professional student skills and behaviours [4–6], and appears to improve patient outcomes [7, 8]. However, integrating effective SBE into health professional curricula can be limited by the high costs and specialized faculty, facility, and technology required [9–11]. Some scholars have called for further SBE cost-effectiveness and cost-comparison studies to inform recommendations for educators working with SBE [11, 12]. These studies might justify SBE integration despite the seemingly high operational expenses, if substantially positive long term, downstream effects and future cost-savings are known.

Alternately, SBE approaches that maintain the “power of simulation” for learners [13] (e.g. deliberate practice [14], observation [14], learning through verisimilitude [13], feedback, debriefing to enable learning from mistakes [13]) but that cost less to implement and maintain, might also alleviate cost barriers to high quality SBE integration. Cook et al. [11] contend that in surgical education, low-cost procedural simulators (e.g. using straws, fruit, and paper) can lead to benefits to learners that resemble those associated with higher cost approaches “because the key elements of the anatomy and the task have been reproduced authentically, even though the apparatus bears little resemblance to the target tissue” (p947). In other words, although the simulator only partly resembles the real task or scenario, the benefits of SBE to learners are maintained because sufficient aspects of the real-world scenario have been authentically replicated in the simulation.

In this research we sought to explore whether student portrayal of patient roles in SBE interactions with their peers might be an innovative, alternative approach to SBE with SPs. Peer simulation, where health professional students portray patient roles in simulated clinical environments, is one alternative to conventional SP-based approaches [15, 16]. Observational studies have investigated health professional students portraying patient roles for the same profession and cohort [15, 17–21], same profession and different cohort [22–25], and for a different profession [26]. Students appear to perceive the interactions as valuable from both a simulated therapist and a SP perspective. Cost savings may exist because students would not need to be recruited or additionally remunerated if portraying patient roles were part of a standard curriculum. This contrasts with high costs for SPs who are paid and have expertise in role portrayal, educational design and feedback [27]. Further, peer simulation might facilitate health professional student development of empathy, communication and professionalism skills [15], as students study and portray patients’ experiences of conditions in the detail required for authentic simulation.

No studies have explored whether health professional students can portray realistic and authentic patient roles in SBE, or if these abilities could improve with training. Investigation of student portrayal abilities, and whether these can be supported by specific education, would provide preliminary data on the potential merit of this approach and provide evidence for the design of larger randomized controlled trials to evaluate the effects, costs, and value of peer simulation.

## Methods

### Study aim

The primary aim of this study was to investigate the abilities of students to portray patient roles in peer simulation. The specific research question for this study was “is completion of a program designed to improve patient role portrayal associated with changes in physiotherapy students’ abilities to portray patient roles?”

### Study design and setting

This study was underpinned by a pragmatic research paradigm. Pragmatic studies address specific practice needs and questions, informed by contexts, to enable feasible and actionable research in real-world settings [28]. Pragmatism prioritizes solving contextual research problems through considering actions, situations, and consequences, and using diverse methods [29].

This research was conducted concurrent with teaching and learning activities of the Western Sydney University (WSU) physiotherapy program. A pragmatic paradigm afforded a study design that was embedded within the normal operations of the program. A repeated measures observational study design explored physiotherapy students’ abilities to portray patient roles before and after completing a pilot peer simulation program. Ethics approval for this study was granted from Western Sydney University Human Research Ethics Committee (project number H10388).

### Participants

Study participants were 2nd year pre-registration Bachelor of Physiotherapy students (PT students) at Western Sydney University enrolled in the required subject *Core Competencies in Physiotherapy*. Students completed a peer simulation program, *Peer Patient*, as part of normal curriculum in 2017. Prior to this, students had completed physiotherapy theory and practical skills subjects, but no SBE or learning activities situated in the clinical environment (clinical placement). A convenience sampling approach was adopted to recruit participants, aligning with the pragmatic study design. Invitations to participate in this study were shared via email and lecture announcement to all students enrolled in the subject *Core Competencies in Physiotherapy* in 2017 (*n* = 57).

### Simulation program

*Peer Patient* (www.peerpatient.com.au) was developed in 2016–17, informed by standards of best practice [30] and guidelines for the design of successful SP programs [31–33]. *Peer Patient* involved completion of two online modules (6 h and 2 h) and three 3-h sessions of classroom-based simulation activities.

Online module 1 aimed to develop patient portrayal skills for SBE. This module required students to review guidelines for peer simulation and participate in written and video activities to develop skills related to preparing for and portraying patient roles. Online module 2 aimed to educate students to portray a specific patient role. This module required students to complete observational activities related to videos of real patients with the condition to be portrayed. This included video capture of a face to face interview regarding the patient’s experience of their condition, and clear demonstration of the impairments and limitations. Students were also required to learn a detailed patient role description, including review of a list of potential questions (from students) with example answers. Students were randomly allocated to portray one out of three patient roles developed. The patient roles included acute care (cardiorespiratory focus), ambulatory care (musculoskeletal focus), and rehabilitation (neurological focus). Students completed both modules in September and October 2017.

Classroom-based peer simulation activities occurred in October 2017. Students working in groups of three adopted one of the peer-therapist, peer-observer, or peer-patient roles, with each student rotating to a different role each week. Sessions comprised preparation (1 h), simulation (1 h), and debriefing (1 h). In the first hour, peer-patients met with an academic in a group of 7–10 to rehearse portrayal, practice physical characteristics, discuss aspects of the role, and receive feedback on portrayal quality and accuracy. Concurrently, peer-therapists reviewed patient-related documentation (medical notes, referral letters) and devised an assessment and management plan. Peer-observers assisted peer-therapists’ preparation. In the second hour, peer-patients and peer-therapists participated in the simulated clinical scenario, while peer-observers assessed peer-therapists’ performances in preparation for debriefing. Peer-observers did not participate in the interaction as an assistant. For the third hour, students stepped out of their roles and completed small group (peer-patient, peer-observer, peer-therapist) and larger group (all students) debriefing activities informed by the SHARP approach [34]. The debrief was focused on the students reflecting on the experience from the perspective of the role they filled. Additional file 1 contains a detailed overview of the *Peer Patient* program, including learning objectives, structure and content.

### Outcome measure

Various instruments exist that provide an assessment of the quality of SP role portrayal [35–37]. However, three members of the research team (SAP, JK, FB) could not identify one instrument that appeared to have adequate content validity for this study (i.e. assessing the quality of patient role portrayal by health professional students). Of the known instruments, items related to SPs providing feedback were not applicable, decision rules for individual item rating were at times unclear, multiple items appeared to assess similar constructs, and items specific to students portraying patient roles (e.g. considered that students have previous medical knowledge, are familiar to their peers, and are unlikely to have prior formal training) were not identified. Therefore, we adapted components of existing instruments and devised a new instrument in three phases.

#### Phase 1 – item articulation

Two members of the research team (SAP, FB) devised the first iteration of the new instrument. Instruments that assess portrayal abilities of SPs were collated [35–37]. Items relevant to students portraying patient roles were extracted, and if required, re-phrased for brevity and context. New items were added to assess the accuracy of the information shared by the peer-patient compared to the patient role outline provided to students. A supplementary guide for assessors outlined the aspects of the portrayal to consider for each item, and examples of high- or low-scoring behaviours.

SAP and FB deliberated on the initial list of items until consensus was achieved on 12 items scored with a 5-point Likert scale of agreeance, and one global rating scale with a 7-point Likert scale of agreeance and free text comments for rating justification. Six of 12 items assessed the accuracy of portrayal with consideration of the role outline provided to students, and six items assessed the quality of portrayal skills.

#### Phase 2 – item refinement

Two members of the research team (SAP, JK) refined the instrument in a series of practice assessments. Two videos of peer simulation interactions that had accompanying patient role outlines were sourced, one from a publicly available site and one from a colleague who had ethics permission to share resources for educational purposes. SAP and JK independently read the patient role outline, watched the video recording of the portrayal, and rated portrayal using the instrument. SAP and JK discussed ratings, comparing scores and reasons for scores. Disagreements were identified and resolved through discussion. Item wording and sequence were clarified, changed or removed to minimise misinterpretations and overlap with related items. The supplementary guide for assessors was refined. Consensus was achieved on a 10-item scale and a global rating scale with a 4-point word-based rating with free text comments for justification.

#### Phase 3 – instrument refinement and training

Three assessors (SAP, DN, JK) met to calibrate their application of the instrument, and where necessary, further refine the instrument and assessor guide prior to starting data collection. Three different videos of peer simulation interactions, with accompanying SP role outlines, were sourced. Assessors independently read patient role outlines, watched video recordings of the portrayal, rated portrayal using the instrument, and compared scores. Differences in item ratings across the three assessors greater than 1-point were identified. Changes were made to the individual item or to the supplementary assessor guide until all three assessors were within 1-point for each item for each video. Consensus was then achieved on a 10-item instrument with a global rating scale (4-point word-based rating with free text comments for justification) and associated supplementary guide that outlined aspects to consider when rating. In addition, detailed examples were provided of high scoring or low scoring portrayal characteristics (Additional file 2). Three scores could be derived from the instrument: 1) a total score for all items, maximum 50, 2) a score for items 1–5 for accuracy relative to the briefing document with a maximum score of 25, 3) a score for items 6–10 for quality of performance with a maximum score of 25.

### Data collection

Students typically portrayed patient roles for each other during routine practical examinations that were video recorded. Ethics approval was obtained to use the video recordings as pre- and post-intervention data for this study. Pre-intervention videos were recorded in July 2017, and post-intervention videos were recorded in December 2017. *Peer Patient* was completed (as part of normal curriculum) in September and October 2017.

In the 10–15 min student examinations, one student portrayed a patient role and one student adopted a therapist role. Patient role outlines (1-page) were written for each examination in a similar format to that in the *Peer Patient* program. Unique scenarios for people and conditions were written specifically for examinations, with patient roles spanning the acute to ambulatory care continuum and across cardiorespiratory, musculoskeletal, and neurological domains of physiotherapy practice. For both examinations, students who portrayed the patient roles were given approximately 5 min to learn the role based on the role outlines. Examinations were filmed from four angles to enable alternative views of the peer patient-therapist interaction, accommodating for the possibility that the primary view might become obscured. A member of the research team (FB) and a research assistant edited all videos to produce a set of four (one primary view and three alternate views) for each patient portrayal to be rated. Videos were given a code, not known to assessors, that removed identifying student information but enabled group (before or after education in portrayal) to be identified.

Three assessors were instructed to a) read the patient role outline, b) watch the primary view of each video in its entirety, c) access additional views of the examination if the primary view was obscured, and d) rate the portrayal using an electronic version of the instrument (hosted as a password protected Google Form). Assessors were blinded to student identity and whether the video was recorded before or after the education intervention. Environment set up and recording angles were identical for pre- and post-intervention video recordings to minimise potential for differentiation by assessors. Ratings were exported and sent to FB to allocate to pre- or post-intervention groups labelled as “1” or “2”, so that all of the research team remained blinded to pre- or post-allocation during data analysis.

### Data analysis

#### Inter-rater reliability

The inter-rater reliability of the instrument was investigated using intraclass correlation coefficients, which were calculated for ratings of total and the two sub-group scores.

#### Primary analysis of interest

The primary analysis of interest for investigating change in patient portrayal abilities was change in total scores for pre- and post-intervention assessments. The mean of assessors’ scores were used as the individual item score for each participant in analysis. Total scores were calculated for ratings of each participant’s before and after program video. The mean and SD of the pre-post total score differences provided the data for a repeated measures analysis. A two-tailed paired t-test (alpha level set at 0.05) was used to determine significance of differences between pre- and post-intervention video scores. The 95% confidence intervals for mean difference calculations used the sample size adjusted t-value.

#### Exploratory analyses

Secondary exploratory analyses were conducted for additional insight into changes in patient portrayal ratings and the rating instrument. Sub-total scores for accuracy of portrayal (based on the briefing outline items 1–5) sub-total scores for quality of portrayal (SP skills, items 6–10), and individual item scores (see Additional file 2) were calculated for each participant’s pre- and post-*Peer Patient* video, and assessed for statistical significance as above.

Microsoft Excel v16.21 for Mac and Stata IC12 were used for all statistical analyses.

## Results

**Fig. 1:**
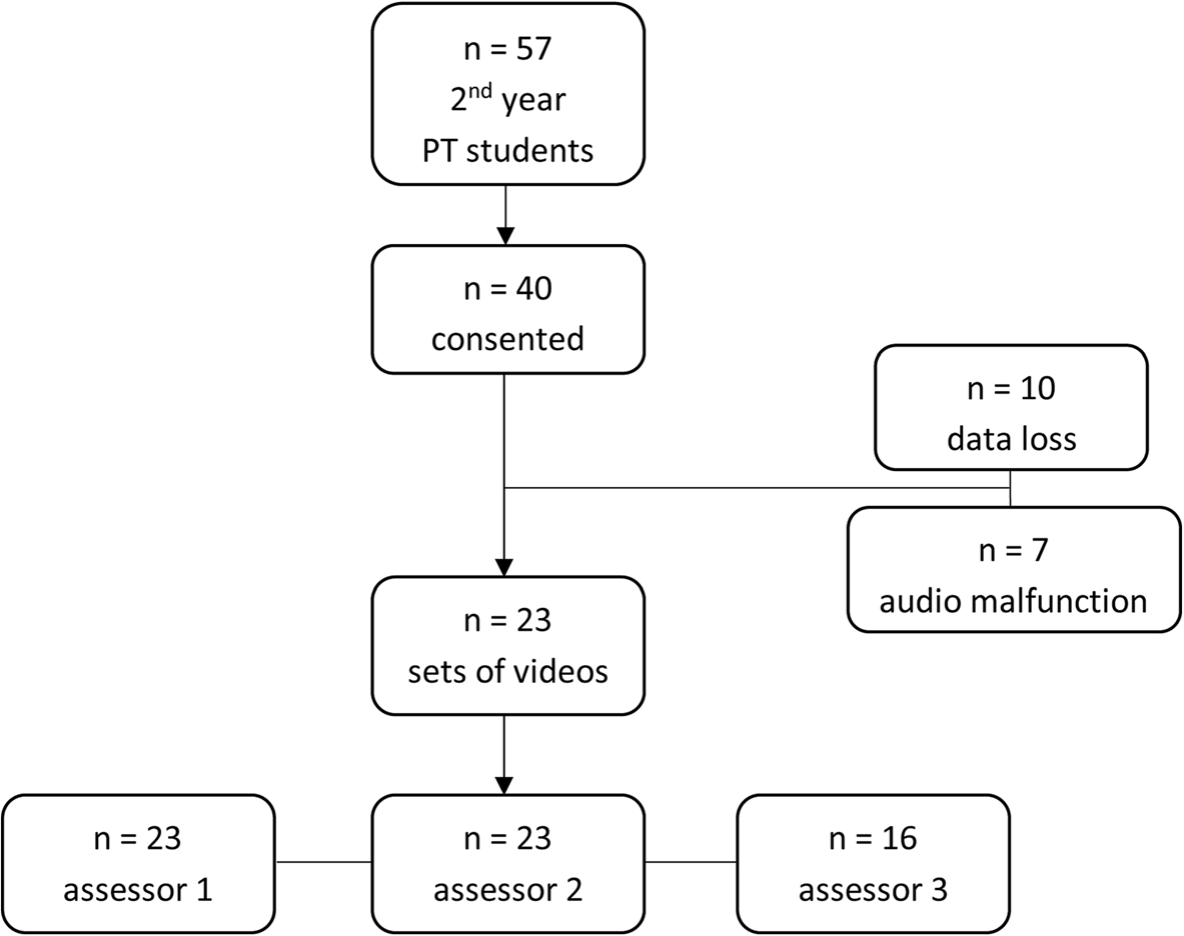
Participant inclusion and data collection flowchart

Of the 57 students who were enrolled in the unit and completed *Peer Patient*, 40 students (70.2%) consented to participate in this study. Twenty-seven participants were female and 14 were male. Ages spanned between 19 and 37 years (mean (SD) 23 (5.07) years, median 20 years). Of the 40 students for whom data was collected, 10 sets of videos were lost while editing, and 7 sets of videos were not assessable due to audio-recording malfunction of the pre or post video. Subsequently, 23 sets of pre- and post-intervention video recordings (58% of target 40 videos) were available for analysis. Two assessors completed ratings for the complete data set of pre- and post-intervention videos (*n* = 23) in August 2018. One assessor completed ratings for a partial data set of pre- and post-intervention videos due to audio malfunction (*n* = 16, 40% of target 40 videos) in November 2018. Consequently, data for the full available data set (*n* = 23) was used in analysis, with data from the third assessor for the subset of 16 cases reported for completeness and transparency. The flow of participant inclusion and data collection is shown in Fig. 1.

### Inter-rater reliability

For the complete data set (*n* = 23), ICC agreement ranged from 0.62 to 0.82 for the total and two sub-group scores for assessor pair 1, indicating a good level of reliability in the use of the instrument. For the primary analysis of interest (total scores), ICC agreement was 0.82 (95%CI [0.62, 0.92]) for the pre-intervention total scores, and 0.66 (95%CI [0.34, 0.84] for the post-intervention total scores. For the sub-total scores for accuracy of portrayal, ICC agreement was 0.80 (95%CI [0.58, 0.91]) for the pre-intervention scores, and 0.62 (95%CI [0.28, 0.82] for the post-intervention scores. For the sub-total scores for quality of portrayal, ICC agreement was 0.77 (95%CI [0.52, 0.90]) for the pre-intervention scores, and 0.68 (95%CI [0.37, 0.85] for the post-intervention scores.

### Primary analysis of interest

The complete data set was assessed for normality using the Shapiro-Wilk test (*p* = 0.77), which justified using parametric tests for the primary analysis of interest. For the complete data set (*n* = 23), the pre-intervention mean (SD) total score was 29.1/50 (8.14), and post-intervention mean total score was 35.6/50 (7.56). A significant difference was observed in favour of the post-intervention mean total scores (mean difference 6.5, 95%CI [1.51–11.45], *p* = 0.01). For the partial data set for which three assessors had rated 16 videos, the pre-intervention mean (SD) total score was 33.3/50 (5.13), and post-intervention mean total score was 37.7/50 (5.56). A significant difference was observed in favour of the post-intervention mean total scores (mean difference 4.4, 95%CI [0.69–8.09], *p* = 0.02).

### Exploratory analyses

#### Sub-total scores

The pre and post-intervention mean sub-total scores (and standard deviations) for accuracy were 13.7 (4.4) and 17.1 (4.2) respectively. A significant difference was observed in favour of the post-intervention mean score (mean difference 3.4, 95%CI [0.69–6.13], *p* = 0.02). For the subset of 16 videos corresponding values were 16.4 (2.9), 18.8 (3.0), and 2.33 [0.21–4.46], *p* = 0.03. The pre and post-intervention mean sub-total scores for quality were 15.4 (4.0) and 18.5 (3.7) respectively. A significant difference was observed in favour of the post-intervention mean score (mean difference 3.1, 95%CI [0.64–5.49], *p* = 0.02). For the subset of 16 videos corresponding values were 16.9 (2.5), 18.9 (3.0), and 2.06 [0.20–3.91], *p* = 0.03.

#### Individual item scores

The individual item pre-program and post-program scores are presented in Table 1. Seven items were significantly higher post-program. The other three items were higher but differences did not attain significance at an alpha level of .05. Similar results were found when data for three assessors and the smaller sample were analysed.

**Table 1:** Individual item scores for complete data set

Items (***n*** = 23)	Pre-program Mean (SD)	Post-program mean (SD)	***p***-value (paired t-test, alpha 0.05)
**Item 1** Accurate history	3.0 (0.8)	3.72 (0.9)	***p*** **= 0.01**
**Item 2** Accurate presenting complaint	3.00 (0.8)	3.74 (0.9)	***p*** **= 0.01**
**Item 3** Accurate ideas, concerns, expectations	2.4 (0.9)	3.11 (1.0)	***p*** **= 0.02**
**Item 4** Accurate physical characteristics	2.6 (1.2)	3.26 (1.0)	*p* = 0.06
**Item 5** Accurate emotion	2.7 (1.1)	3.26 (0.9)	***p*** **= 0.04**
**Item 6** Appropriate appearance	4.4 (0.9)	4.63 (0.6)	*p* = 0.32
**Item 7** Character embodiment	2.6 (0.9)	3.30 (1.0)	***p*** **= 0.02**
**Item 8** Appropriate information sharing	2.9 (0.9)	3.70 (0.9)	***p*** **= 0.01**
**Item 9** Stayed “in character”	3.0 (1.1)	3.61 (1.1)	*p* = 0.07
**Item 10** Appropriate improvisation	2.52 (1.0)	3.26 (1.0)	***p*** **= 0.02**

## Discussion

This is the first study to investigate physiotherapy students’ abilities to portray patient roles in SBE. Student completion of educational activities designed to improve patient role portrayal appears to lead to measurable improvements in portrayal abilities. Further evaluation of peer simulation as a potential substitute for other SBE and clinical education models appears to be warranted.

Scores for student portrayal of patient roles were significantly higher after completion of *Peer Patient*. In the second practical examination (after the program), the key elements that characterize a higher standard of patient portrayal (accuracy of portrayal and quality of portrayal) were more prevalent. Given the nature of the constructs assessed by the instrument (accuracy and quality of portrayal), and that the education program was designed to specifically educate these constructs, it is not unreasonable to conclude that the improvements observed post-program may indeed be due to this program.

Exploratory analyses showed that multiple aspects of patient portrayal contributed to the improvements observed in total scores. The sub-categories of accuracy of students’ portrayal (to the specific patient scenario which students were briefed on), and the quality of the portrayal (SP skills) were rated higher post-program. The practice guides that were used to inform the design of this program specify that remembering role details (i.e. accuracy), and being able to portray role details consistently and realistically (i.e. quality) are necessary for high quality SP-based education that engages learners [32]. These exploratory analyses provide further evidence that student portrayal was improved across multiple constructs.

Average student portrayal scores improved from below an “adequate” rating in the pre-program examination, to above an “adequate” rating in the post-program examination. While the study design limits conclusions that can be drawn regarding whether the portrayal was sufficiently realistic for students to engage in SBE (i.e. whether the “power of simulation” [13] was maintained), from the educators’ point of view, students did not portray sufficiently adequate patient roles in the first examination (pre-program), but did in the second examination (post-program). This observation supports the notion that students might be able to portray sufficiently realistic patient roles that enable student engagement. Further research comparing student to lay-person portrayal of patient roles, and data regarding student perspectives on the realism of peer patients would provide insight into whether peer simulation sufficiently maintains the “power of simulation” for learners.

The size of the improvement observed in students’ role portrayal abilities (6.5 points on a 50-point scale) is likely to be pedagogically meaningful, in addition to being statistically significant. Students scored reasonably well before completing the program (pre-intervention mean 29.1/50), and improved by a significant amount. In addition, the change in total scores was greater than half a standard deviation (SD). Norman et al. [38, 39] contend that the threshold of discrimination for minimally important differences in health-related instruments is approximately half a SD. The change observed in this study was approximately 80% when considering the pre-intervention SD of 8.14. Pragmatic factors restricted preparation activities to be considerably shorter and less resourced than ideal, which, if more preparation time had been provided, may have resulted in an even more dramatic effect. As such, it is not unreasonable to conclude that the magnitude of the difference reflects meaningful improvement for the purposes of student portrayal of patient roles in SBE.

The pragmatic nature of this study meant that contextual factors influenced the design and implementation of this study. A predetermined curriculum required several decisions, assumptions, and design features to be considered by the researchers. The timing of the assessments of patient portrayal (practical examinations scheduled for nearly 2 months prior to and after training), who was able to assess portrayal (SAP, DN, JK), the different experiences and backgrounds of the assessors, and how portrayal could be recorded (audio-visual limitations) are some of the factors that could not be altered for this study. While these factors might be limitations of the work, they were reasonable to adopt for this study which intended to collect *preliminary* data to inform arguments for or against further exploration of this approach in larger and more robust study designs. Other confounding factors that may have influenced results include student concurrent learning, and time alone. It is unlikely that the pre-existing and other curriculum that students were completing across the study period (neuroanatomy, pathophysiology, and clinical pharmacology) would have significant influence on portrayal abilities, as the portrayal rating instrument assessed specific aspects relevant to learning objectives of *Peer Patient*, and these conditions were not taught in any other aspect of the curriculum. In addition, external experiences of students across the 6-month study period such as being a patient themselves, seeing a family member with the target condition, or conducting their own research, might have influenced student abilities.

Conversely, these pragmatic study design factors also provide opportunities for improvements in the conditions under which students were asked to portray patient roles. For example, in the practical examinations where portrayal was assessed, students were afforded minimal time (likely less than 5 min) to learn the role brief and did not have video references or educator-supported training and feedback on their performance. These factors are widely endorsed as important for SP-based education [30]. The structure of the patient role briefs might also be improved. In this study, role briefs were kept consistent to those that had always been used in the curriculum to enable similar conditions before and after the *Peer Patient* program. Despite these conditions, students still improved by a significant margin, to above an adequate level of portrayal performance. There is potential for even stronger development of SP skills with ongoing refinement of materials that support student portrayal of patient roles.

Strengths of this study design include achieving assessor blinding to group (before or after participation in *Peer Patient*), and designing similar conditions for student portrayal of patient roles in both before and after *Peer Patient* role assessments. A limitation of this study is that within the constraints of pragmatic curriculum management, considerable data were lost due data management processes and audio malfunction, which reduced the sample size. Nevertheless, these significant results justify further investigation of this approach. Future research might consider student development of expertise in other aspects of SP practice, including scenario design and feedback [27]. In the examinations used for data collection, students were patients and examined (as therapists) on the same day. A limitation of this approach may be that anxiety associated with an examination may have influenced their portrayal. However, the first and second examinations adopted an identical process in an attempt to minimise the influence on validity of pre- and post-program data. Additionally, consideration of the perspectives of real patients’ on the realism of students portraying patient roles may be worthy of further investigation, given real patients can have different priorities and perspectives to healthcare personnel regarding simulated healthcare scenarios [40].

Given that students appear to be portraying patient roles well, and even better after targeted training, a formal trial appears to be warranted that includes comparisons between the cost-effectiveness of peer simulation, SBE with SPs, and informal role play. Further data regarding students’ experiences and perspectives of peer simulation (exploring perceived changes to communication, empathy and professionalism abilities, and exploring whether the “power of simulation” exists for learners) might also inform improvements to curriculum design and implementation recommendations. A randomised controlled trial that directly compares and contrasts different peer simulation training activities would provide data regarding the specific design features that support students to develop patient portrayal skills. Cost-effectiveness studies that consider both student attainment of learning outcomes and the costs required to achieve these outcomes [11] would be valuable to inform guidelines for integrating this approach to SBE in health professions education.

## Conclusions

Educational activities designed to improve patient role portrayal appear to be associated with improvements in physiotherapy students’ abilities to portray patient roles in SBE. Peer simulation might be a viable alternative to SBE with SPs. Further investigation of the cost-effectiveness of peer simulation in larger studies with more robust research designs appears warranted.

## Supplementary Information

**Additional file 1:.** Appendix 1. Peer Patient overview

**Additional file 2:.** Appendix 2: Rating tool

## Data Availability

The datasets used and/or analysed during the current study are available from the corresponding author on reasonable request.

## Abbreviations

SBE: Simulation-based education
SP: Simulated patient
