# Supplementary material for: Physiotherapy students can be educated to portray realistic patient roles in simulation: a pragmatic observational study
Source: BMC Med Educ. 2020 Nov 26;20:471. doi: 10.1186/s12909-020-02382-0 (PMC7689969; doi:10.1186/s12909-020-02382-0)
Supplement: Supplementary file 1 — Additional file 1:. Appendix 1. Peer Patient overview [file 12909_2020_2382_MOESM1_ESM.docx]

| **Curriculum component** | **Learning objectives** | **Structure and content** |
| --- | --- | --- |
| Online module 1: How to… | ​By the conclusion of module 1 learners will:   - Outline the principles of acting as described in the Stanislavski techniques and the Strasberg’s Method - Relate the Stanislavski techniques and the Strasberg’s Method to filling the role of a simulated patient - Analyse the effective uses of the human voice as a means of communicating ideas and feelings, and demonstrate communication of emotions through verbal and physical means. - Demonstrate an ability to mirror movement, both normal and abnormal movement patterns. - Demonstrate basic competency in acting, voice and speech, and movement to realistically replicate a simulated patient case.​ | 1. Guidelines for Your Peer Simulation Experience   Video with captions (approx. 6 minutes providing overview of guidelines to make the most of simulation experience   1. Prepare for the Simulated Patient Role   10 videos (approx. 1 minute each) introducing SP concepts, spoken by an experienced SP   1. The Craft of Acting for Patient Simulation   Article presenting acting skills and techniques, including the Method acting technique (interspersed with pictures)   1. Observe and Portray Movement Patterns   Three activities including review of videos, activities to identify physical characteristics seen on video, and guided activities for practicing physicality   1. Observe and Portray Emotions   Four activities targeting identification of emotions and embodiment in physicality |
| Online module 2: Patient cases | By the conclusion of module 2 learners will:   - Recall the components of a simulated case that are important to access and review prior to filling the role of simulated patient, and understand why the components are essential for filling the role. - Recall the information about their character for the simulation based learning experience - Outline how the pathophysiological changes associated with the disease process lead to the signs and symptoms, impairments, activity limitations, participation and the patients’ sense of self. - Be able to accurately portrayal the movement and emotions of a specific patient case in a simulation based classroom experience. - Realistically improvise in character of the specific patient case in a simulation based classroom experience. | 1. Watch the patient video (physiotherapy assessment) and take notes 2. Complete observational notes activity 3. Read the script, presented in the template of character, health issue, simulation environment 4. Watch the patient video (impact of health condition) 5. Complete question/response activity |
| Face-to-face hour 1 (preparation) | By the conclusion of the three hour simulation, learners will be able to:   - Gather relevant information from a medical record for a patient presenting to physiotherapy with common cardiorespiratory/musculoskeletal/neurological dysfunction in an acute cate/rehabilitation/ambulatory care setting. - Formulate and conduct a patient interview for a patient presenting to physiotherapy with common cardiorespiratory/musculoskeletal/neurological dysfunction in an acute cate/rehabilitation/ambulatory care setting. - Formulate and conduct a physical examination for a patient presenting to physiotherapy with common cardiorespiratory/musculoskeletal/neurological dysfunction in an acute cate/rehabilitation/ambulatory care setting. - Interpret and analyse assessment findings to formulate a prioritised problem list and definition of client needs in an acute cate/rehabilitation/ambulatory care setting. - Communicate effectively for the purpose of gaining informed consent and engaging a person in a therapeutic alliance for a person-centred clinical assessment. - Apply safe work practices, professional behaviours and self-management strategies for a clinical context. - Critically self-reflect and evaluate the performance of the therapist and client in a clinical context. | Peer patients:  10 minute review of patient role details  5 minute discussion led by tutor about case details  5 minute rotating role play led by tutor  10 minutes physical examination practice in pairs  20 minutes environment set up (costume, props)  Peer therapists:  Review clinical paperwork provided by tutor  Plan interview and physical examination  Peer therapists:  Familiarise with observational activities (assessment tool)  Review clinical paperwork  Support peer therapists to formulate plan |
| Face-to-face hour 2 (simulation) |  | 60 minute simulated clinical interaction  Students working in groups of three, supervised by a tutor  Utilisation of time-in / time-out process to allow students deliberate practice |
| Face-to-face hour 3 (debrief) |  | 1. 5 minutes independent reflection time and de-role-ing 2. 25 minutes guided debriefing and feedback in small groups of three 3. 25 minutes of guided debrief in the whole group (approx. 18 students) |
