# Supplementary material for: Physiotherapy students can be educated to portray realistic patient roles in simulation: a pragmatic observational study
Source: BMC Med Educ. 2020 Nov 26;20:471. doi: 10.1186/s12909-020-02382-0 (PMC7689969; doi:10.1186/s12909-020-02382-0)
Supplement: Supplementary file 2 — Additional file 2:. Appendix 2: Rating tool [file 12909_2020_2382_MOESM2_ESM.docx]

**Student as peer-patient (PP) – portrayal rating tool**

**Video code:** ____________ **Completed by: ­_________________**  **Date: ___/___/_______**

**Objective**: to assess students’ abilities to portray simulated patient roles, informed by rating their accuracy to the role outline and the quality of portrayal.

**Ratings**: 1 = very poorly OR did not portray this characteristic

2 = poorly

3 = adequately

4 = well

5 = very well

*Please circle your rating for each statement:*

| **Item** | **The peer patient, on portraying the patient role…** | **Rating** | | | | |
| --- | --- | --- | --- | --- | --- | --- |
| 1. | …reported the patient’s history accurately | 1 | 2 | 3 | 4 | 5 |
| 2. | …reported the patient’s present complaint/issue accurately | 1 | 2 | 3 | 4 | 5 |
| 3. | …reported the patient’s ideas, concerns and expectations accurately | 1 | 2 | 3 | 4 | 5 |
| 4. | …displayed accurate physical characteristics and movements | 1 | 2 | 3 | 4 | 5 |
| 5. | …displayed accurate emotion | 1 | 2 | 3 | 4 | 5 |
| 6. | ...displayed an appropriate appearance | 1 | 2 | 3 | 4 | 5 |
| 7. | ...embodied the information and character | 1 | 2 | 3 | 4 | 5 |
| 8. | ...shared appropriate breadth of information | 1 | 2 | 3 | 4 | 5 |
| 9. | ...stayed "in character" | 1 | 2 | 3 | 4 | 5 |
| 10. | ...improvised appropriately | 1 | 2 | 3 | 4 | 5 |

*Please circle an overall rating of the* *peer patient portrayal****,*** *relative to the* ***expected performance*** *of the patient:*

| ***Not Adequate*** | ***Adequate*** | ***Good*** | ***Excellent*** |
| --- | --- | --- | --- |

*Please explain in a few words why you gave this rating.*

**Assessor guide:**

For all items, consider the consistency of the peer patient’s portrayal of these elements, with the information and instructions provided in the **patient role outline**:

| **Item** | **Explanation** | **Example** |
| --- | --- | --- |
| 1. | Accurately remembered and reported history and character information, including explicit and/verbatim responses (i.e. an opening statement, specific answers to specific questions).  *NB: refers to the patient’s history prior and separate to the history related to their present complaint (assessed in item 2).* | Consistency with past medical history, medications, social history, etc. |
| 2. | Accurately remembered and reported information about the patient’s present complaint or issue, including explicit and/verbatim responses (i.e. an opening statement, specific answers to specific questions).  *NB: does not refer to the patient’s history prior and separate to their present complaint (assessed in item 1).* | Consistency with current problems and limitations, current pain levels, mechanism of injury, previous treatment, etc. |
| 3. | Accurately remembered and reported the patient’s underlying ideas, main concerns, and expectations. | Consistency with current beliefs about problems, main concerns about health, worries etc. |
| 4. | Accurately displayed physical characteristics and movements, including physical characteristics and movements that weren’t explicitly outlined but would be expected for the patient being portrayed* | Consistency with patient’s limb movement, speaking, walking, etc.  *e.g. consistent performance in a movement test performed in different way to that explicitly outlined (e.g. a lower score might be obtained if patient was able to sit on low chair but was only explicitly instructed to move knee no greater than 90 degrees flexion) |
| 5. | Accurately displayed emotion, however it manifests in the interaction. | Consistency of voice intonation changes, facial expressions, eye contact, fidgety hand movements, etc. |
| 6. | For elements that patient was given advance notice on, appearance was appropriate to the patient role. | Consistency of clothing, props, make-up |
| 7. | Integrated, embodied and reported information in ways that were appropriate to that which might be expected.  *NB: does* ***not*** *assess the accuracy of the content, but the nature in which it was portrayed.* | A lower score for this item might be obtained if a peer patient knew the correct information, but shared it in a way that was disjointed, confusing, or inconsistent with what would be expected of the patient (e.g too little, or too much sharing). |
| 8. | Shared and withheld information consistent with what is expected of the patient, considering the way a particular question was asked, or request was made for the patient. | A lower score for this item might be obtained if a peer patient over-shared information or over-performed in response to poor quality questions and instructions, or under-shared information or under-performed in response to higher quality questions and instructions. |
| 9. | Consider instances when the peer patient came “out” of the patient role during the portrayal. | A lower score for this item might be obtained if a peer patient giggled when the patient would not have probably done this. |
| 10. | Consider the occasions when the peer patient appeared to improvise to remain in character. That is, engaged in conversation and responded to questions and instructions in a way that was consistent with that of this patient, but had not been explicitly outlined. | A higher score for this item might be obtained if a peer patient talked about children’s hobbies, but this was not explicitly outlined. |
